# Genetic Variants on Chromosome 8q24 and Colorectal Neoplasia Risk: A Case-Control Study in China and a Meta-Analysis of the Published Literature

**DOI:** 10.1371/journal.pone.0018251

**Published:** 2011-03-24

**Authors:** Mian Li, Yanhong Zhou, Peizhan Chen, Huan Yang, Xiaoyan Yuan, Kazuo Tajima, Jia Cao, Hui Wang

**Affiliations:** 1 Key Laboratory of Nutrition and Metabolism, Institute for Nutritional Sciences, Shanghai Institutes for Biological Sciences, Chinese Academy of Sciences, Graduate School of the Chinese Academy of Sciences, Shanghai, People's Republic of China; 2 Department of Hygienic Toxicology, College of Preventive Medicine, Key Lab of Medical Protection for Electromagnetic Radiation, Ministry of Education of China, Third Military Medical University, Chongqing, People's Republic of China; 3 Division of Epidemiology and Prevention, Aichi Cancer Center Research Institute, Nagoya, Japan; Ohio State University Medical Center, United States of America

## Abstract

Previous studies have found that common genetic variants on chromosome 8q24 are associated with the risk of developing colorectal neoplasia. We conducted a hospital-based case-control study, including 435 cases and 788 unrelated controls to investigate the associations between common variants on 8q24 and the risk of colorectal cancer in a Chinese population. We also evaluated the association of rs6983267 with colorectal neoplasia in the published literature via a meta-analysis study. We found that rs6983267 was significantly associated with the risk of colorectal cancer in the Chinese population, with an adjusted odds-ratio (OR) for the GT heterozygotes and GG homozygotes of 1.30 (95% CI  = 0.98–1.71, P = 0.069) and 1.66 (95% CI  = 1.18–2.34, P = 0.004), respectively, compared to the TT homozygotes, with a P-trend value of 0.003. No association was found for the other three loci (rs16901979, rs1447295 and rs7837688). In the meta-analysis of the published genetic association studies, the rs6983267 variant was found to be associated with an increased risk of colorectal neoplasia. The heterozygous GT carriers showed a 20% increased risk of colorectal neoplasia (OR  = 1.20, 95% CI  = 1.16–1.25; random effects model) with a summary OR for homozygous GG carriers of 1.39 (95% CI  = 1.32–1.48; random effects model) compared to the TT genotype carriers. We found no significant differences between the association of rs6983267 and colorectal cancer and colorectal adenomas. In summary, our study confirms that the variant rs6983267 is a risk factor for colorectal neoplasia in various populations, including the Chinese population.

## Introduction

Colorectal cancer (CRC) is the third most common cancer and the fourth most frequent cause of cancer death worldwide [1]. The lifetime risk in Western European and North American populations is around 5% [2]. Among the risk factors and causes for CRC, inherited genetic factors account for approximately 35% of the disease etiology [2]. Many genetic factors, such as mutations of critical genes (*e.g.* APC, MLH1, MSH2, TGFBR2 and SMAD4) have been identified [3]. However, many factors that increase the susceptibility to CRC, but with low penetrance need to be explored. In the past few years, several genome-wide association studies (GWAS) have identified novel loci that are associated with CRC risk, such as variants on 11q23 [2], 8q24 [4], [5], [6], 10p14 [7], 8q23.3 [7], 15q13.3 (HMPS) [8] and SMAD7 [9]. However, the extent to which these genetic factors contribute to the disease has not been established.

Among these loci, variants on 8q24 have shown strong evidence of an association with the risk of CRC in different populations. Haiman *et al* genotyped six variants in 1,124 individuals with invasive CRC and 4,573 controls that had been previously identified as having an underlying risk for prostate cancer due to alterations on 8q24 [10] and found that one variant, rs6983267, was also significantly associated with colorectal cancer [4]. Similarly, Tomlinson *et al* conducted a genome-wide association study of 550,000 tag SNPs in 930 familial colorectal cancer patients and 960 controls and found that rs6983267 had the strongest association with CRC risk [5]. Analyses based on 1,477 individuals with colorectal adenoma and 2,136 controls suggested the possibility that this locus is involved in tumor initiation rather than progression [5]. SNP rs10505477, which maps about 5.86 kb centromeric to rs6983267 and has a high linkage disequilibrium with rs6983267, was also found to be associated with CRC risk, and has been implicated across many cohort and case-control studies [5], [11], [12], [13]. It has also been reported that five different haplotype blocks at the 8q24 region have been identified, and only those loci located between the 128.47 Mb and the 128.54 Mb region (*e.g.* rs6983267, rs10808556 and rs10505477) were associated with CRC risk. The variations in the other regions were found to be associated with prostate, breast and ovarian cancers [14].

The constantly improving standard of living in China has brought about extensive changes to the lifestyle and diet of the average Chinese citizen over the past three decades, and has led to a corresponding increase in the incidence and mortality of CRC in China [15]. The steady increase in the incidence and mortality of CRC has made the disease one of the leading causes of death in China. However, few studies have examined the genetic factors that influence the risk of CRC in the Chinese population. We herein report the results of a case-control study that we performed to investigate the allelic variants on 8q24 and their effect on CRC risk in a Chinese population. To further investigate the association of variant rs6983267 with the CRC risk, a systemic review of the literature published about the locus was undertaken, and a meta-analysis was conducted.

## Materials and Methods

### Study populations

Most of the participants in the study have been described previously [16]. In brief, a total of 478 CRC patients and 838 controls aged between 30 and 80 years old were enrolled between 2001 and 2003 from three hospitals (Xi'nan Hospital, Xinqiao Hospital and Daping Hospital) in Chongqing, China. The subjects were genetically unrelated ethnic Han Chinese from Chongqing and the surrounding regions served by these hospitals, including parts of Sichuan, Yunnan and Guizhou provinces in southwest China, which are adjacent to Chongqing. The recruitment followed the Japan, Korea and China Colorectal Cancer Collaboration Group guidelines. All patients had been histopathologically diagnosed with primary CRC within the past 6 months, and had not received any treatment. The medical records of the patients were thoroughly checked and for those who were suffering from ileocecal junction tumors or anal canal tumors and those with any of the following conditions: i. recurrence of CRC; ii. familial adenomatous polyposis (FAP); iii. hereditary nonpolyposis colorectal cancer (HNPCC); iv. other tumors; v. severe digestive tract diseases lasting over 2 years; vi. diabetes, fatty liver, hepatic cirrhosis, metabolic syndrome or severe cardiovascular diseases were excluded from the study. One or two controls matched to each eligible case based on age (±5 years), sex and residence were selected. The controls were recruited from non-CRC patients seen in the Departments of General Surgery, Orthopedics or Trauma who were admitted for trauma, bone fractures, appendicitis, arthritis, or varicose veins. Patients with tumors, severe digestive tract diseases lasting over 2 years, diabetes, fatty liver, hepatic cirrhosis, metabolic syndrome or severe cardiovascular diseases were also excluded from the control group.

A 5-mL peripheral venous blood sample was obtained from each subject after written informed consent was obtained. Each participant was personally interviewed by trained interviewers to complete a Semi-quantitative Food Frequency Questionnaire (SQFFQ), which collected demographic information and information about dietary, and smoking habits (current smoker, former or never smoker), alcohol use (more or less than 15 g/day, according to the recommended level of daily alcohol consumption suggested by the China Health Care Association) and other lifestyle factors [16]. After quality control procedures were completed for both SQFFQ and DNA samples, a total of 435 patients and 788 controls of Han ethnicity were finally included in the study.

### Ethics statement

The study was approved by all of the ethics committees of the participating hospitals (“Ethics Committee of Xi'nan Hosipital”, “Ethics Committee of Xinqiao Hosipital” and “Ethics Committee of Daping Hosipital”). All of the samples were collected with a written informed consent provided by the participants, and all protocols were approved by the human research ethics committees of the participating hospitals.

### Selection of SNPs on 8q24

Genome-wide association studies and gene-based candidate studies have confirmed that chromosome 8q24 is a susceptibility region for CRC. It has also been shown that specific loci on 8q24 are associated with specific cancers [14]. Among these loci are two SNPs, rs6983267 and rs10505477, which were separately found to be associated with CRC risk [5], [17]. These two SNPs are in high linkage disequilibrium (LD) in the Chinese population (r^2^ = 0.95) according to the Hapmap database, and they were also found to be associated with an increased risk of prostate, breast and ovarian cancers in many population studies [4], [18], [19]. We chose to further study the association of rs6983267 with colorectal cancer in the Chinese population. Another 3 SNPs, rs16901979, rs1447295 and rs7837688, which were previously found to be associated with prostate cancer, were also selected to evaluate their association with colorectal cancer risk in the Chinese population [14], [20].

### DNA isolation and genotyping

Genomic DNA was extracted from 2.5-mL of whole blood with a Promega DNA Purification Wizard kit according to the manufacturer's instructions. The genotyping methods have been reported previously [16]. Three SNPs (rs16901979, rs1447295 and rs7837688) were genotyped using the Applied Biosystems SNPLex system (Applied Biosystems Incorporated, California, USA) together with 45 other loci including previously reported loci on CYP2E1 [16]. Loci were submitted online to ABI Inc for probe design and synthesis. The OLA (oligonucleotide ligation assay), purification, and PCR reactions were performed on an Eppendorf 5333 Mastercycler, and allele inspection was performed on an ABI 3130xl Gene Analyzer. The SNP information was collected using Data Collection Software version 3.0, and data were analyzed by the GeneMapper Software version 4.0. Another locus (rs6983267) was genotyped using a TaqMan® SNP Genotyping Assay (Applied Biosystems Incorporated, California, USA) on a 7900HT Fast Real-Time PCR System (Applied Biosystems Incorporated, California, USA). A total of 10% of the samples were randomly selected for duplication for these loci to assess the reproducibility of the genotyping calls and a more than 99% concordance rate was found.

### Meta-analysis of the rs6983267 locus in subjects with colorectal neoplasia

**Figure 1:**
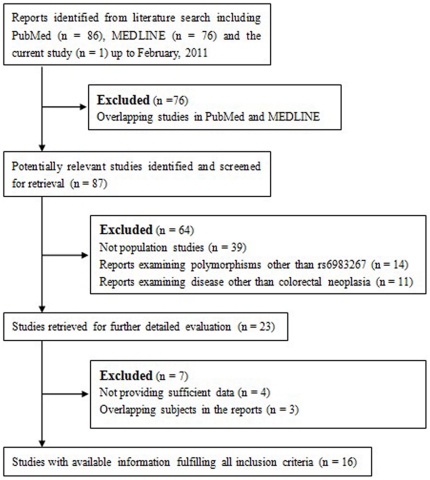
The flow chart for the selection of studies and specific reasons for exclusion of studies from the meta-analysis.

In order to explore the association between rs6983267 and the risk of colorectal neoplasia, a meta-analysis was conducted. We performed a comprehensive and systematic bibliographic search updated to February of 2011 based on the MEDLINE and PubMed databases. We used the terms “8q24” and “rs6983267” in combination with “colorectal neoplasia”, “colorectal cancer” or “colorectal adenoma” to search the database to identify the studies regarding the association between the rs6983267 polymorphism and the risk of colorectal cancer. References were also checked to identify any missing studies. The details of the studies were thoroughly examined in order to exclude potentially overlapping data. If the same participants were used in different papers, only the largest and most complete study was included here. The studies included were those that provided data about rs6983267 and the risk of colorectal cancer or colorectal adenomas and could be case-control, cohort or cross-sectional studies that were reported in English. The studies included should also provide sufficient data about the frequency of the genotypes. Individual authors were also contacted for further data when the criteria were not met. The flow chart that tracks the selection process for the studies and the reasons for exclusion is presented in Figure 1.

For each study, the following information was recorded: first author, publication year, study design, study location, study population/ethnicity, total number and sources of cases and controls, sub-group and disease categories, total number of cases and controls, and the allele frequency in the study. When there were sub-group studies described in the paper (such as those stratified by ethnicity, study stage, *etc*), they were considered individually. Pooled allelic effects were estimated both under a fixed effects model and a random effects model, and the pooled OR with its 95% CIs was used as the summary measurement.

### Statistical analysis

The prevalence of each of the alleles was measured in cases and controls. The χ^2^ test (for categorical variables) and Student's t-test (for continuous variables) were used to evaluate the differences in demographic characteristics and selected variables. Hardy-Weinberg Equilibrium (HWE) was assessed by the χ^2^ test (1 degree of freedom (d.f)). The common homozygote was used as the reference to calculate the genotype-specific odds ratio (OR) and its 95% confidence intervals (CI) with or without adjustment for age, sex, smoking status and alcohol use under the unconditional Logistic regression statistic model. The statistical powers of the study were calculated using the Power software under the assuming of two sided test with alpha level is 0.05 with the log-additive genetic model [21], [22].

For meta-analysis, the pooled OR and its 95% CI were calculated using the standard inverse variance weighting method for the fixed effects model and the DerSimonian-Laird method for the random-effects model. Heterogeneity between studies was assessed using the Cochrane Q-test in combination with the I^2^ statistic. Publication bias was graphically represented by funnel plotting and was assessed both by Egger's linear regression [23] and Begg's rank correlation tests [24]. Statistical analyses were undertaken using the R Software with the SNPassoc and Meta packages (http://www.r-project.org/).

## Results

The characteristics of cases and controls are given in Table 1. There were more patients with a daily average alcohol intake of more than 15 g/day (P = 0.011) in the neoplasia patients, as has been indicated previously [15]. In addition, the cases were slightly older than the controls (P = 0.017). Other potential confounders were not significantly different between the cases and controls (Table 1). The genotyping results of the selected SNPs in the CRC cases and controls are shown in Table 2. The overall call rate of each SNP was more than 98%, and no SNP deviated from Hardy-Weinberg equilibrium (P>0.05, Table 2).

**Table 1:** The characteristics of the participants from a Chinese population.

Variance	Controls (N = 788)	Cases (N = 435)	P-value
Age (years)^a^	51.73±11.29	53.49±12.88	0.017
Sex			0.972
female	351 (44.5%)	195 (44.8%)	
male	437 (55.5%)	240 (55.2%)	
Smoking status			0.547
former and never	488 (61.9%)	261 (60.0%)	
current	300 (38.1%)	174 (40.0%)	
Alcohol use (>15 g/day)			0.011
yes	152 (19.3%)	112 (25.7%)	
no	636 (80.7%)	323 (74.3%)	

a Age was presented as the mean ± SD (years).

**Table 2:** An association study of 8q24 loci and colorectal cancer risk.

SNP	Genotype	Controls(N %)	Cases(N %)	Crude OR(95% CI)	Adjusted OR^a^(95% CI)	P value^a^	P-trend^a^	Call rate	HWE-testP-value
rs16901979	CC	403 (51.5)	219 (50.7)	1	1			99.3%	0.66
	AC	321 (41.0)	171 (39.6)	0.98 (0.76–1.26)	0.95 (0.74–1.22)	0.706			
	AA	58 (7.4)	42 (9.7)	1.33 (0.87–2.05)	1.30 (0.84–2.00)	0.243	0.550		
rs6983267	TT	256 (32.6)	111 (25.8)	1	1			99.4%	0.61
	GT	392 (49.9)	219 (50.9)	1.29 (0.98–1.70)	1.30 (0.98–1.71)	0.069			
	GG	138 (17.6)	100 (23.3)	**1.67 (1.19–2.35)**	**1.66 (1.18–2.34)**	**0.004**	**0.003**		
rs1447295	CC	567 (72.5)	294 (67.9)	1	1			99.3%	0.39
	AC	202 (25.8)	127 (29.3)	1.21 (0.93–1.58)	1.22 (0.94–1.59)	0.140			
	AA	13 (1.7)	12 (2.8)	1.78 (0.80–3.95)	1.73 (0.78–3.87)	0.180	0.062		
rs7837688	GG	587 (75.8)	308 (71.0)	1	1			98.8%	1.00
	TG	175 (22.6)	117 (27.0)	1.27 (0.97–1.67)	1.26 (0.95–1.65)	0.103			
	TT	12 (1.6)	9 (2.1)	1.43 (0.60–3.43)	1.38 (0.57–3.32)	0.479	0.089		

a Adjusted for age, sex, alcohol use and smoking status.

Statistically significant associations were obtained only for the previously reported SNP rs6983267 (Table 2). Compared to the TT homozygotes, the GT heterozygotes showed a marginally increased risk of colorectal cancer (adjusted OR = 1.30, 95% CI = 0.98–1.71; P = 0.069). However, the GG homozygotes showed a significant, 66% increased risk of colorectal cancer (adjusted OR = 1.66, 95% CI = 1.18–2.34; P = 0.004). The locus was also found to be associated with a gene-dose response relationship for an increased risk for CRC (P-trend = 0.003). The statistical power for the association of SNP rs6983267 and CCR in our study was 0.839 for the observed OR. We found no statistically significant interactions between rs6983267 and age, sex, smoking status or alcohol use. No other locus was found to be significantly associated with the CRC risk (Table 2). However, the statistical powers for the three loci were relatively lower (0.290 for rs16901979, 0.702 for 1447295 and 0.316 for rs7837688 for the observed ORs).

**Figure 2:**
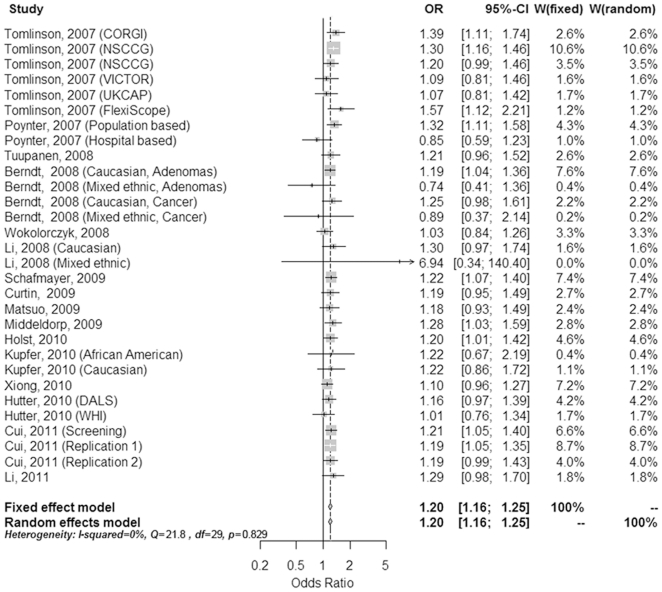
A forest plot of the association of colorectal neoplasia with rs6983267 heterozygosity (G/T vs. T/T).

**Figure 3:**
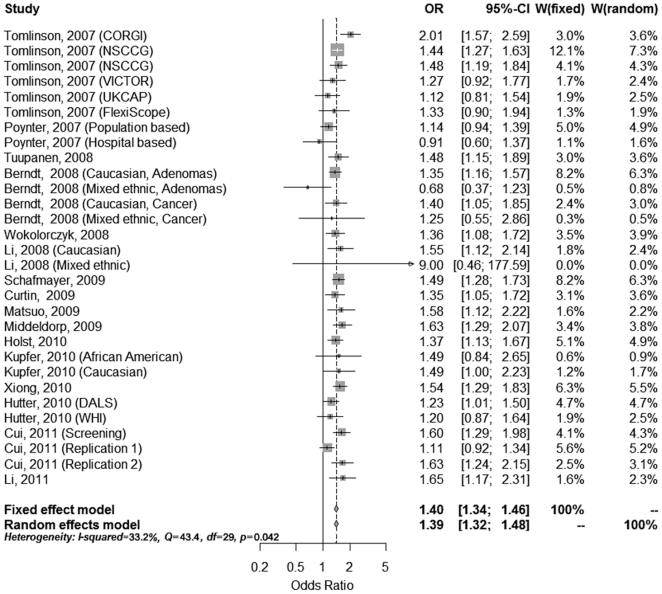
A forest plot of the association of colorectal neoplasia with rs6983267 homozygosity (G/G vs. T/T).

For the meta-analysis regarding the association between colorectal cancer risk and the variant rs6983267, we identified twenty-three reports regarding the association between rs6983267 and colorectal neoplasia. After detailed evaluation, sixteen studies including a total of 36,761 cases and 38,901 controls were used for the meta-analysis (Table 3) [5], [11], [12], [13], [19], [25], [26], [27], [28], [29], [30], [31], [32], [33], [34]. Four studies lacked sufficient data about the genotype frequency [4], [14], [35], [36] and three studies contained overlapping participants with the included reports [37], [38], [39], and were therefore excluded from the analysis (Figure 1). For rs6983267, the summary odds ratio (SOR) was 1.20 (95% = 1.16–1.25) under the fixed effects model and the random effects model for the GT heterozygotes compared to the TT homozygotes (Figure 2). There was no significant heterogeneity between the studies (Q = 21.77, df = 29, P = 0.829; I^2^ = 0%), and no evidence of a significant publication bias was found (Begg's test, P = 0.630; Egger's test, P = 0.465). For the GG homozygotes, we noted that there was a significant increase in colorectal neoplasia, with the SOR of 1.40 (95% CI = 1.33–1.46) under the fixed effects model and 1.39 (95% CI  = 1.32–1.48) under the random effects model compared to the TT carriers (Figure 3). However, significant heterogeneity was found among these studies (Q = 43.39, df = 29, P = 0.042; I^2^ = 33.2%). However, there still no significant publication bias (Begg's test, P = 0.762; Egger's test, P = 0.783). Sensitivity analyses were also performed by removing the individual studies sequentially, and we found that none individual studies dramatically affected the overall polled ORs.

**Table 3:** Studies included in the meta-analysis of the association of rs6983267 with colorectal neoplasia.

Study(First author, year)	Country and ethnicity	Study design	Sub-group study and disease category	Cases	Controls	Ref
Tomlinson, 2007	UK, Caucasian	Population based case-control	CORGI, cancer	620	960	[5]
	UK, Caucasian	Population based case-control	CORGI, adenomas	407		
	UK, Caucasian	Nested case-control	NSCCG, cancer	4361	3752	
	UK, Caucasian	Nested case-control	NSCCG, cancer	1901	1079	
	UK, Caucasian	Nested case-control	VICTOR, cancer	1072	415	
	UK, Caucasian	Nested case-control	UKCAP, adenomas	607	765	
	UK, Caucasian	Population based case-control	FlexiScope, adenomas	463	411	
Poynter, 2007	USA and Canada, Mixed ethnic	Population based case-control	Cancer	1339	2191	[11]
	USA and Canada, Mixed ethnic	Hospital based case-control	Cancer	288	502	
Berndt, 2008	USA, Caucasian	Nested case-control	Adenomas	2569	2779	[12]
	USA, Mixed ethnic	Nested case-control	Adenomas	151	194	
	USA, Caucasian	Nested case-control	Cancer	538	1644	
	USA, Mixed ethnic	Nested case-control	Cancer	71	165	
Tuupanen, 2008	Finland, Caucasian	Population based case-control	Cancer	996	1012	[13]
Wokolorczyk, 2008	Poland, Caucasian	Hospital based case-control	Cancer	779	1910	[19]
Li, 2008	USA, Caucasian	Population based case-control	Cancer	527	679	[25]
	USA, Mixed ethnic	Population based case-control	Cancer	34	42	
Curtin, 2009	UK and USA, Caucasian	Population based case-control	Cancer	1069	1040	[26]
Matsuo, 2009	Japan, Japanese	Hospital based case-control	Cancer	481	962	[27]
Schafmayer, 2009	Germany, Caucasian	Population based case-control	Cancer	2712	2713	[28]
Middeldorp, 2009	Netherlands, Caucasian	Population based case-control	Cancer	995	1340	[29]
Kupfer, 2009	USA, African American	Hospital based case-control	Cancer	1795	2378	[30]
	USA, Caucasian	Hospital based case-control	Cancer	399	367	
Holst, 2010	Sweden, Caucasian	Population based case-control	Cancer	1786	1749	[31]
Xiong, 2010	China, Chinese Han	Hospital based case-control	Cancer	2124	2124	[32]
Hutter, 2010	USA, Caucasian	Population based case-control	Cancer	1461	1813	[33]
	USA, Caucasian	Nested case-control	Cancer	614	633	
Cui, 2011	Japan, Japanese	Population based case-control	Screening stage, cancer	1583	1898	[34]
	Japan, Japanese	Population based case-control	Replication 1,cancer	3099	1777	
	Japan, Japanese	Population based case-control	Replication 2, cancer	1485	819	
Li, 2011	China, Chinese Han	Hospital based case-control	Cancer	435	788	

To determine whether the association between variant rs6983267 and colorectal neoplasia could differ for colorectal adenomas and colorectal cancer, we examined the association between the variant and colorectal cancer and the variant and colorectal adenomas. Among the fourteen identified reports, two examined the association between the variant and colorectal adenomas, with a total of 4,197 cases and 5,109 controls [5], [12]. All of the reports studied the association between rs6983267 and colorectal cancer, with a pool of 32,564 total cases and 34,752 controls. From the meta-analysis, we found that the SORs for GT heterozygotes and GG homozygotes compared to the TT homozygotes were similar in magnitude to those found for colorectal adenomas and total colorectal cancer (Table 4). In the subgroup analysis by ethnicity, we found that the variant rs6983267 was associated with an increased risk of colorectal neoplasia both in the Asian populations (9,207 cases and 8,368 controls) and the Caucasians (23,818 cases and 25,021 controls), although there was a slight different of the pooled OR between the Asian populations and for the Caucasians (Table 4).

**Table 4:** The results of the meta-analysis of the association of rs6983267 with colorectal neoplasia.

Category		Genotype(Cases/Controls)	Fixed effects model	Random effects model	Q value/df	P value for Q test	I^2^	P value of Begg's test	P value of Egger's test
Disease	Neoplasia	TT (8,334/10,088)	1	1					
		GT (17,583/18,400)	1.20 (1.16–1.25)	1.20 (1.16–1.25)	21.77/29	0.829	0%	0.630	0.465
		GG (10,770/10,372)	1.40 (1.33–1.46)	1.39 (1.32–1.48)	43.39/29	0.042	33.2%	0.762	0.783
	Adenomas only	TT (862/1,253)	1	1					
		GT (2,110/2,538)	1.20 (1.08–1.34)	1.21 (1.04–1.41)	6.10/4	0.192	34.4%	1.000	0.868
		GG (1,225/1,318)	1.36 (1.21–1.53)	1.32 (1.00–1.74)	15.09/4	0.005	73.5%	0.624	0.712
	Carcinomas only	TT (7,537/9,024)	1	1					
		GT (15,471/16,335)	1.20 (1.15–1.25)	1.20 (1.15–1.25)	16.09/25	0.912	0%	0.843	0.647
		GG (9,478/9,356)	1.41 (1.34–1.48)	1.41 (1.33–1.49)	31.46/25	0.174	20.5%	0.708	0.584
Ethnic group	Caucasian	TT (4,670/6,041)	1	1					
		GT (11,953/12,566)	1.21 (1.16–1.27)	1.21 (1.16–1.27)	11.24/17	0.844	0%	0.791	0.591
		GG (7,195/6,414)	1.42 (1.35–1.49)	1.42 (1.35–1.49)	16.37/17	0.498	0%	0.677	0.745
	Asian	TT (3,276/3,338)	1	1					
		GT (4,382/3,898)	1.18 (1.10–1.26)	1.18 (1.10–1.26)	1.46/5	0.918	0%	0.573	0.371
		GG (1,533/1,131)	1.45 (1.32–1.59)	1.48 (1.28–1.70)	10.66/5	0.059	53.1%	0.573	0.369

## Discussion

Variants on 8q24 (position 128.14 Mb to 128.62 Mb) were found to be associated with various types of cancer. It was reported by Ghoussaini *et al* that there are five different haplotype blocks within this region. The rs6983267 locus and its highly correlated locus, rs10505477 (r^2^ = 0.95 in CHB population for Hapmap database), which are located between a 128.47 and 128.54 Mb region, have been found to be associated with prostate, colorectal and ovarian cancers [14]. Loci outside this region were also found to be associated with prostate or breast cancers, but not colorectal cancer [14]. Our study also found a significant association between rs6983267 and colorectal cancer in the Chinese population. However, no significant association for the other three loci, which are located outside the 128.47 and 128.54 Mb region, was identified in the Chinese population. As indicated in our previous study and in other studies, alcohol use is a risk factor for colorectal cancer. In the stratification analysis, we found a trend toward a stronger association in the participants without a drinking habit (<15 g/d), however, we found no statistically significant interactions between the variant rs6983267 and alcohol use. We also did not find any significant interactions between rs6983267 CRC risk and other potential confounders. These findings were consistent with other studies indicating that the association between rs6983267 and colorectal cancer may not modified by the age at diagnosis [11], [12], [25], [27], sex [12], [25], [27], family history of CRC [11], [12], [25], [27], smoking status [27], or alcohol use [27]. However, the current study is a hospital based case-control study and the number of participants was relatively small, which may have led to spurious results. Whether the association between rs6983267 and colorectal neoplasia was affected by the confounders needs further investigation.

In the meta-analysis of rs6983267 and colorectal neoplasia, sixteen eligible papers that examined the risk of colorectal carcinoma, including two that also reported the association between the locus and colorectal adenomas risk were examined. We found that the GT heterozygotes had a 20% (95% CI = 1.16–1.25; random effects model) and the GG homozygotes showed a 39% (95% CI = 1.32–1.48; random effects model) increased risk of colorectal neoplasia. The SORs determined for colorectal neoplasia were similar in magnitude to those observed for colorectal cancer and colorectal adenomas (Table 4). These findings indicated that rs6983267 may play a similar role in the etiology of both colorectal cancer and colorectal adenomas. Although the frequency for the risk allele G was different in different populations (0.487 in CEU, 0.394 in CHB and 0.301 in JPT according to Hapmap database), we also found that the strength of the association between rs6983267 and colorectal neoplasia were similar between the Caucasian and Asian populations (Table 4), which was consistent with a previous meta-analysis study conducted by Hutter et al concerning the association between this locus and CRC risk [33].

Although the variant rs6983267 had been found to be associated with an increased susceptibility to colorectal neoplasia, whether the rs6983267 SNP is the causal locus is still uncertain. Several recently published studies have shown that rs6983267 is located within a transcriptional enhancer region and affects a binding site for TCF4 (also called TCF7L2), a transcription factor which is activated in most CRCs. TCF4 interacts with β-catenin to activate the transcription of Wnt target genes, which are part of a key pathway involved in CRC initiation [40], [41]. It has been found that the rs6983267 G allele has an approximately 1.5-fold stronger enhancer activity and increased affinity for TCF4 compared to the T allele. And Pomerantz et al. demonstrated that a DNA fragment region containing the variant shows a long-range physical interaction with the MYC promoter located ∼330 kb downstream using a chromatin conformation capture (3C) technique [40]. These findings indicate the biological mechanism(s) that potentially underlie the role of this non-protein-coding risk variant, and indicated that rs6983267 may be a causal variant for the susceptibility to CRC.

There are several potential limitations to the current study. First, for a hospital-based case-control study, subjects were recruited based on their outcome (with CRC or without CRC) rather than their exposure. The potential CRC risks for the controls are immeasurable, and may have caused a cause bias in studying the etiology of the disease. It could also have led to spurious results in the gene/environmental interaction studies. Second, the sample size was relatively small. We cannot exclude the possibility there is an association between rs16901979, rs1447295 and rs7837688 and colorectal cancer in the Chinese population due to the inadequate statistical power of our study (0.290 for rs16901979, 0.702 for 1447295 and 0.316 for rs7837688, respectively), although many other studies have also reported no significant association for these loci with CRC in other populations [12], [14], [26], [28]. Accordingly, the null interaction between the confounders (alcohol use, smoking status, sex, age) and rs6983267 may also be due to the low power of small sample size. Third, there are at least five different cancer susceptibility regions on the 8q24 “desert” as reported by Ghoussaini et al [14]. Only the loci in the third region such as rs6983267 and rs10505477, were found to be significantly associated with CRC, and evidence from the in vitro experiment indicated that rs6983267 may be the causal locus, although we cannot exclude the possibility that other functional variants which are highly correlated with rs6983267 also lead to an increased risk of CRC. This study evaluated the representative loci from different regions on 8q24 and their association with CRC risk in an Asian (Han Chinese), which has been not well studied for these variants so far. It is valuable to conduct a fine-mapping study for the third region to identify other potential causal variants that are associated with CRC risk in a larger Chinese population.

In summary, we observed a statistically significant association between SNP rs6983267 on 8q24 and the risk of colorectal cancer in a Chinese population. Moreover, a meta-analysis of previous studies conducted in different populations confirmed the association between this locus and colorectal neoplasia risk in different populations and ethnic groups. These results provide a more complete picture of the role of this polymorphism in the risk of colorectal neoplasia, and may give genetic insight into possible strategies for prevention of colorectal neoplasia.
